# Combined Effect of Colloids and SMP on Membrane Fouling in MBRs

**DOI:** 10.3390/membranes10060118

**Published:** 2020-06-06

**Authors:** Dimitra Banti, Manassis Mitrakas, Georgios Fytianos, Alexandra Tsali, Petros Samaras

**Affiliations:** 1 Laboratory of Technologies of Environmental Protection and Utilization of Food By-Products, Department of Food Science and Technology, International Hellenic University, GR-57400 Thessaloniki, Greece; gfytianos@gmail.com (G.F.); tsalialex@gmail.com (A.T.); samaras@food.teithe.gr (P.S.); 2 Laboratory of Analytical Chemistry, Department of Chemical Engineering, Aristotle University of Thessaloniki, GR-54124 Thessaloniki, Greece; manasis@eng.auth.gr

**Keywords:** membrane fouling, particle size distribution, SMP, EPS, synthetic wastewater, domestic wastewater

## Abstract

Membrane fouling investigations in membrane bioreactors (MBRs) are a top research issue. The aim of this work is to study the combined effect of colloids and soluble microbial products (SMPs) on membrane fouling. Two lab-pilot MBRs were investigated for treating two types of wastewater (wwt), synthetic and domestic. Transmembrane pressure (TMP), SMP, particle size distribution and treatment efficiency were evaluated. Chemical Oxygen Demand (COD) removal and nitrification were successful for both kinds of sewage reaching up to 95–97% and 100%, respectively. Domestic wwt presented 5.5 times more SMP proteins and 11 times more SMP carbohydrates compared to the synthetic one. In contrast, synthetic wwt had around 20% more colloids in the mixed liquor with a size lower than membrane pore size (<400 nm) than domestic. Finally, the TMP at 36 days reached 16 kPa for synthetic wwt and 11 kPa for domestic. Therefore, synthetic wwt, despite its low concentration of SMPs, caused severe membrane fouling compared to domestic, a result that is attributed to the increased concentration of colloids. Consequently, the quantity of colloids and possibly their special characteristics play decisive and more important roles in membrane fouling compared to the SMP—a novel conclusion that can be used to mitigate membranes fouling.

## 1. Introduction

Shortcomings of existing conventional biological processes for wastewater treatment, such as poor effluent quality, particulates in their outflow, low volumetric and organic loading rates, high sludge production, high footprint, reduced nitrogen nitrification, low efficient in dye removal and high requirements for disinfection of the wastewater treated, have forced the community to find new treatment methods, such as those membrane bioreactors (MBRs) could offer [1,2,3,4]. Despite the many advantages of MBRs, membrane fouling caused by interactions between activated sludge suspension and membrane still remains their main disadvantage, limiting their expanded application [3,5,6].

Fouling could be attributed to the adhesion of activated sludge components, such as soluble microbial products (SMPs), extracellular polymeric substances (EPSs) and colloids on the surface of the membrane or within the inner surface of pores, causing deposition in the form of biofilm and/or complete pore blockage, respectively [7,8]. SMPs and EPSs are affected from various parameters, such as temperature and season. According to Mesquita et al. (2010) [9] when the temperature is low their concentration is decreased, whereas temperature fluctuations cause biomass stress and increase SMP and EPS.

Membrane fouling is caused due to the presence of dissolved inorganic and/or organic compounds in the mixed liquor. Soluble microbial products (SMPs) are the main components of dissolved organic matter (DOM) that are common in wastewater. During biological wastewater treatment, DOM could influence kinetic activity and flocculating properties of activated sludge [10]. According to previews studies, the relative contribution of DOM to membrane fouling in MBRs varied between 26% and 52% [11,12]. SMPs and EPSs are secretions of microorganisms with high molecular weight, and a three-dimensional, gel-like form. Furthermore, they are highly hydrated molecules often presenting charged biofilm matrix, where microorganisms are incorporated and stabilized [13]. SMP are either released into the liquor during cell lysis or secreted during substrate metabolism and decrease during cells creation [7]. EPSs, on the other hand, are cell-bound and consist of a dynamic double-layer structure, which is divided into loosely bound EPS (LBEPS) and tightly bound EPS (TBEPS) [14].

Soluble organic components, fine compounds, colloids and soluble salts found in wastewater are responsible for membrane fouling [12], participating in various fouling mechanisms. The following six dominant mechanisms are most reported: (a) pore blocking, (b) cake formation, (c) concentration polarization, (d) organic adsorption, (e) inorganic precipitation and (f) biological fouling [12]. Regarding the classification of particulate matter, settleable solids are greater than 100 μm, supra-colloidal solids vary from 1 to 100 μm and colloidal solids from 0.001 μm (10 Å) to 1 μm, whereas dissolved solids do not exceed 10 Å. Among aquatic colloids, corrosion products, silt and clay, precipitated crystals, colloidal silica and sulfur, precipitated iron and aluminum compounds could be detected [12]. A behavior related to inorganic particles has also been observed in high molecular weight (MW) organics; for instance, polysaccharides, peptidoglycans, proteins humic aggregates and their clusters (e.g., cellular debris) [15]. During membrane fouling due to colloids, initial pore blocking occurs and cake formation follows [12,16]. Microfiltration (MF) membranes efficiently reject any suspended solids larger than membrane pore size to the mixed liquor, as a result of their microporous structure, while the colloids and any soluble matter smaller than the membrane pore size may penetrate through the membrane pores, adhere onto membrane surface or clog the pores [17].

Regarding the comparison of synthetic and domestic wastewater, limited research has been conducted until today. According to Villain et al. (2014) [18] who have dealt with this subject, the MBR of synthetic wastewater treatment contained 50 mg/L SMP proteins and 60 mg/L SMP carbohydrates, while in comparison the MBR of domestic wastewater treatment contained 40 mg/L SMP proteins and 0 mg/L SMP carbohydrates. However, the other operating parameters of the two units (for synthetic and domestic treatment) were not similar in order to conclude in comparative SMP values, with an mixed liquor volatile suspended solids (MLVSS) value of 9.1 g/L in the case of synthetic wastewater, and 7.6 g/L in the case of domestic wastewater. It should be noted that mixed liquor suspended solids (MLSS) and mixed liquor volatile suspended solids (MLVSS) are used to determine the concentration of microorganisms. MLVSS constitute the biologically active part of the solids, determined by igniting the MLSS and they depict the organic fraction of MLSS. Comparing membrane fouling for the two types of wastewater, the hydraulic resistances were calculated, and it was found that removable/reversible fouling was 3% higher on the membrane used for synthetic wastewater treatment.

Therefore, research to date has shown that both the dissolved organic matter (DOM) in the form of SMP and the colloids play significant roles in membrane fouling. However, a comparison of different wastewater types in order to evaluate the synergistic contribution of SMP and colloids on membrane fouling is yet to be carried out. The aim of this work is to cover this research gap, and to study the combined effects of colloids and SMP on membrane fouling in MBRs. For this reason, two MBR units were used to treat two different types of wastewater, evaluating the variation of SMP and colloids value, along with the effluent quality characteristics.

## 2. Materials and Methods

### 2.1. Membrane Bioreactor Set-up and Operating Conditions

**Figure 1:**
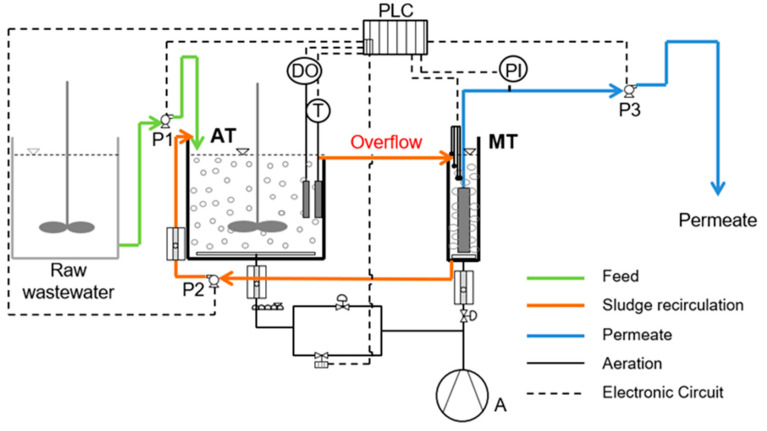
Flow diagram of the membrane bioreactor (MBR) units. AT: aeration tank (20 L), MT: membrane tank (5 L), PLC: programmable logic controller, CO: air compressor, DO: dissolved oxygen controller, PI: pressure indicator, P1: raw wastewater feed pump, Ρ2: activated sludge recirculation pump, Ρ3: effluent pump.

Two similar lab-pilot scale MBR experiments were conducted successively, using a commercial hydrophilic membrane module in both MBRs. They consisted of an aeration tank (AT) and a membrane tank (MT), as shown in Figure 1. A flat sheet microfiltration (MF) membrane module was immersed in the MT (Table 1). The cake layer from the membrane’s surface was scoured with strong aeration supplied from the bottom of the MT with air sparging rate of 15 L/min. The permeate flux was intermittent with an operation step of 10 min followed by a relaxation step of 2 min. The working volume of the AT in both MBRs was 20 L, and dissolved oxygen (DO) was adjusted to 2.5 ± 0.5 mg/L by regulating air flow. The working volume of the MT was 5 L. No sludge was abstracted during the MBR operation in either case. The MBRs were located in a closed and protected room, where the temperature was controlled at 24 ± 2 °C in order not to affect the process.

The first membrane bioreactor was constantly fed with synthetic wastewater that simulates domestic wastewater, hereafter called synthetic wwt. The synthetic wastewater contained the following chemicals [1,19]: 500 mg/L glucose, 500 mg/L corn starch, 200 mg/L NH_4_Cl, 56 mg/L peptone, 53 mg/L KH_2_PO_4_, 18 mg/L MgSO_4_·7H_2_O, 7.32 mg/L MnSO_4_·H_2_O and 1.1 mg/L FeSO_4_·7H_2_O. In addition, NaHCO_3_ at a concentration of 240 mg/L was used to maintain the pH between 7.0 and 7.5 in both bioreactors. The Chemical Oxygen Demand (COD) value of synthetic wastewater was ranged at 447 ± 68 mg/L. At the beginning, the bioreactors were filled with activated sludge from a full-scale municipal wastewater treatment plant of Thessaloniki, and during the following two weeks the wastewater was gradually acclimated to synthetic wastewater.

The second membrane bioreactor was constantly fed with real domestic wastewater, hereafter called domestic wwt, originating from the municipal wastewater treatment plant of Thessaloniki (Greece) after primary treatment (screening–sand separation–primary settling). The domestic MBR had an average COD value of 496 ± 71 mg/L. MLSS average concentration for both MBRs was maintained at 4500 ± 500 mg/L. The mean food/microorganisms (F/M) ratio was equal to 0.16 ± 0.03 g COD/g MLSS/d for the synthetic wwt, and to 0.24 ± 0.03 g COD/g MLSS/d for the domestic wwt.

A dissolved oxygen meter, three peristaltic pumps, an air compressor, a pressure indicator and a thermometer were contained in the MBRs. The MBR was controlled by a PLC system (Eutech Instruments, Singapore, Republic of Singapore) through SCADA software (Simantec, Siemens, Version 14). Various parameters were measured for online control and evaluation of the system’s operation, such as temperature, DO, transmembrane pressure (TMP), influent, recirculation and effluent fluxes. Aiming to draw safe conclusions, the MBR units operated twice treating synthetic and domestic wastewater and both times the MBRs showed similar values and trends. This article presents in detail the results of the second period of operation of the MBRs.

### 2.2. Critical Flux Determination

Critical flux represents an important operation parameter of MBRs, and it is defined as the flux below which reversible fouling occurs, while the TMP remains almost stable [20,21]. The critical flux of the MBRs was evaluated as described by van der Marel et al. (2009) [21] using the flux-step method. The critical flux of the synthetic MBR was found to be slightly greater than 16.4 Lmh, and therefore the MBR was adjusted to operate in subcritical flux conditions, at 16.4 Lmh. Thus, the influent (Q_in_), recirculation (Q_r_) and effluent fluxes (Q_eff_) were 1.8, 3.6 and 1.8 L/h, respectively. The critical flux of the domestic MBR was found to be slightly greater than 20.9 Lmh, and therefore the MBR was adjusted to operate in subcritical flux conditions, at 20.9 Lmh. Consequently, the domestic MBR operated with influent, recirculation and effluent fluxes of Qin = 2.3 L/h, Qr = 4.6 L/h, Qeff = 2.3 L/h, respectively.

### 2.3. Determination of SMP, LBEPS, TBEPS and Physicochemical Parameters

Regarding the SMP fractions, a physical extraction method was adapted from Hwang et al. (2010) [22] and Banti et al. (2017) [1], as described below. A mixed liquor sample (35 mL) taken from the aerated tank of the MBR units was dewatered by centrifugation at 4000 g for 5 min. After that, the supernatant liquid phase from the centrifugation was filtered through 0.45 μm membrane filters, and the filtrate was utilized for the determination of SMP [23]. A solution of 0.05% NaCl was used for resuspension of the sludge pellet in the centrifugal tube at 50°C which was subsequently shared with a vorter mix (G-560, Scientific Industries, Inc., Bohemia, NY, USA) for 1 min. The mixed liquor was then centrifuged at 4000 g for 10 min, and the supernatant was used for determination of loosely bound EPS (LBEPS). To extract further TBEPS, the sludge pellet was resuspended in 0.05% NaCl solution to a final volume of 35 mL. The sludge suspension was heated at 60 °C in a water bath for 30 min; then centrifuged at 4000 g for 15 min; and the supernatant liquid phase was used for the determination of TBEPS fraction.

The SMP, LBEPS and TBEPS extracts were further analyzed to determine their protein concentrations in triplicate and polysaccharide concentrations in duplicate. Polysaccharide concentrations were measured following the photometric method proposed by Dubois et al. (1956) [24] and protein concentrations were measured following the modified Lowry method [25]. Protein concentrations were calibrated with bovine serum albumin (BSA, Sigma Aldrich) and polysaccharides with glucose (Panreac).

Wastewater characteristics and effluent quality parameters (COD, N-NH_4_, N-NO_3_, total N) were determined with Hack–Lange LCK kits, along with a DR-2800 spectrophotometer. It should be noted that chemical oxygen demand (COD) is a method for estimating the amount of oxygen would be depleted from a body of receiving water as a result of bacterial action. Total suspended solids were measured according to standard methods [26].

### 2.4. Determination of Particle Size Distribution

A dynamic light scattering (DLS) instrument (Brookhaven Instruments Corporation, Holtsville, NY, USA) was used for measuring particle size distribution, for the components with size less than 1 μm, coming from the mixed liquor and the effluent of the MBRs. Pre-filtration of the samples with Whatman Puradisc syringe filters of 3.5 μm pore size was performed in line with DLS measurement sample preparation method. Aiming to results of high precision, five measurements were carried out automatically for each sample. Brookhaven Instruments Corporation (BIC) Particle Solutions Software^®^ and Microsoft Office software^®^ were used to process the results and determine the percentage distributions, respectively.

## 3. Results

### 3.1. Wastewater Treatment Efficiency

**Figure 2:**
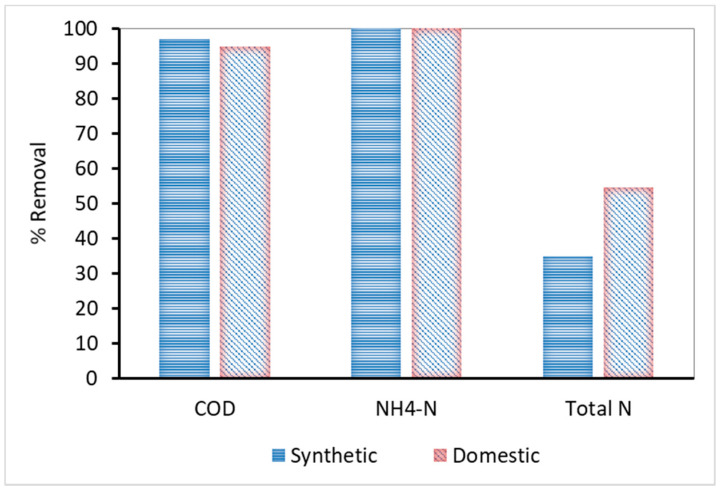
COD, NH_4_-N and total N percentage removal for synthetic and domestic wastewater.

The characteristics of influent wastewater, both synthetic and domestic, and the effluent characteristics are presented in Table 2, whereas Figure 2 shows their removal percentage. According to the results, the COD removal was particularly high, reaching 97% and 95% for synthetic and domestic wastewater, respectively. The lower removal of COD by the domestic MBR is mainly attributed to the presence of non-biodegradable organic compounds in domestic wastewaters. The nitrification process via conversion of NH_4_-N to NO_3_-N was successful for both kinds of sewage, reaching 100% removal. It should be noted that neither MBR unit included a denitrification section, and therefore the nitrogen removal was solely attributed to biosynthesis.

### 3.2. Production of SMP, LBEPS and TBEPS Proteins and Carbohydrates

Table 3 compares the mean, min and max values of SMP, LBEPS and TBEPS in the form of proteins and carbohydrates for the two types of wastewater. It is observed that much greater concentrations of SMP were produced in domestic wwt. The domestic wwt had 5.5 times more SMP proteins and 11 times more SMP carbohydrates compared to the synthetic wwt, which are attributed to higher F/M ratio and lower residence time in domestic MBR (Table 2). Furthermore, the increased values of SMP proteins in domestic wwt are related to the lower COD/N ratio, compared to the synthetic wwt. It is worth noting that the mean F/M ratio was equal to 0.24 ± 0.03 g COD/g MLSS/d for the domestic wwt, and 0.16 ± 0.03 g COD/g MLSS/d for the synthetic wwt. Moreover, COD/N ratio in domestic wwt was equal to 6.6, whereas in synthetic ones it was equal to 9.7.

Table 4 compares the SMP and EPS proteins/carbohydrates ratios for both types of wastewater. It is concluded that proteins are consistently higher than carbohydrates for SMP and EPS for both types of wastewater. Specifically, protein concentrations of SMP and EPS of domestic wwt are 4–6 times higher than carbohydrates, whereas in synthetic wwt they are 6–10 times higher, as it was expected. Additionally, it was observed that the SMP proteins/carbohydrates ratio and LBEPS proteins/carbohydrates ratio in synthetic wwt are almost double those of domestic wwt. This can be attributed to the fact that the synthetic wwt MBR unit had a longer hydraulic retention time, equal to 13.9 h, compared to the domestic wwt, where it was equal to 10.9 h.

**Figure 3:**
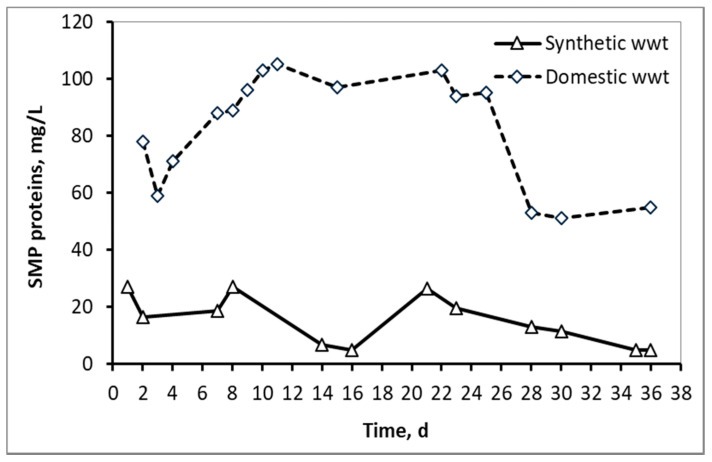
SMP concentration in the form of proteins in the mixed liquor of synthetic and domestic wastewater.

**Figure 4:**
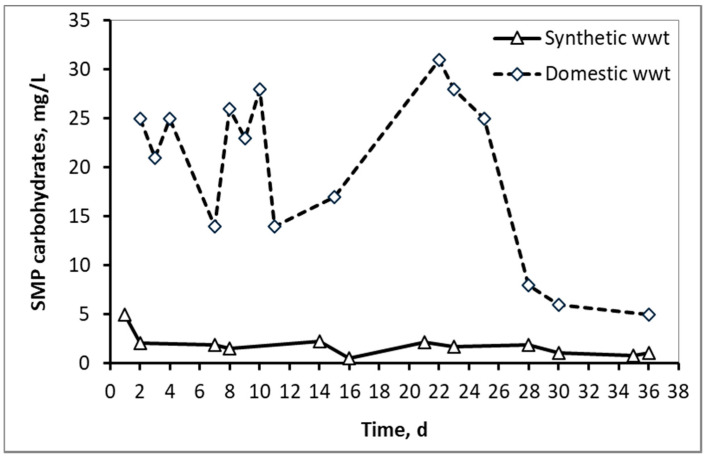
SMP concentration in the form of carbohydrates in the mixed liquor of synthetic and domestic wastewater.

Figure 3 and Figure 4 compare the fluctuations of SMP proteins and carbohydrates respectively between the two types of wastewater, as a function of the MBR operation time. In the case of domestic wwt, greater fluctuations of SMP concentrations are observed, due to the variation at the inflow COD, as the MBR received urban real time wastewater from a full-scale wastewater treatment plant.

**Figure 5:**
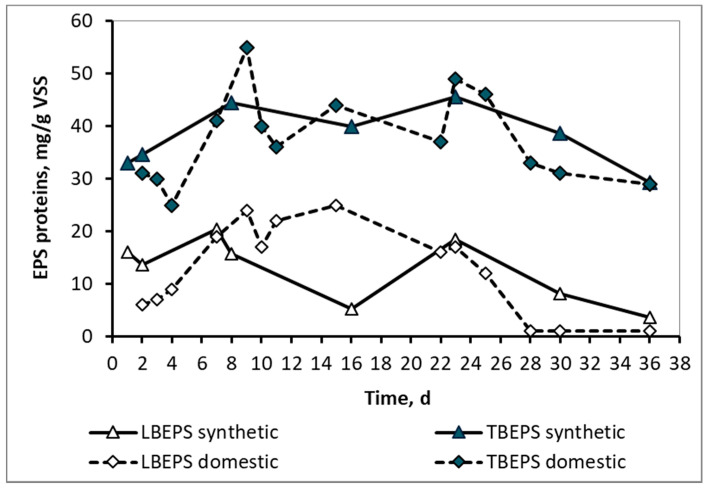
Loosely bound EPS (LBEPS) and tightly bound EPS (TBEPS) concentrations in the form of proteins for the mixed liquor of synthetic and domestic wastewater.

**Figure 6:**
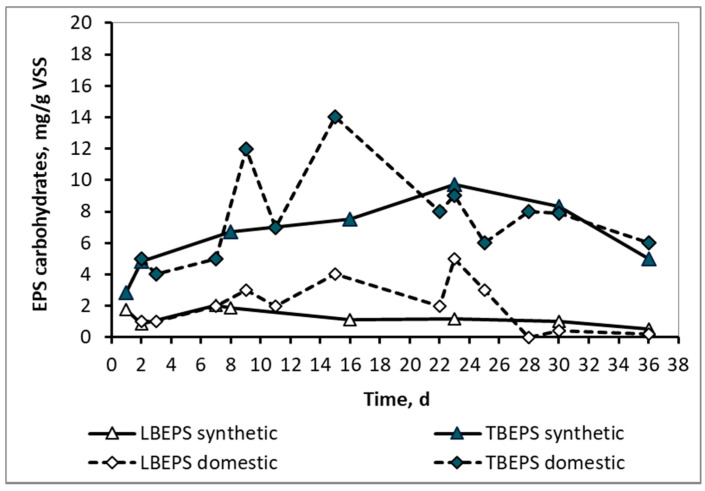
LBEPS and TBEPS concentrations in the form of carbohydrates for the mixed liquor of synthetic and domestic wastewater.

In contrast to the SMP, the LBEPS and TBEPS concentrations, either in the form of proteins or in the form of carbohydrates, as shown in Figure 5 and Figure 6 respectively, ranged at about the same levels for both synthetic and domestic wwt. This result is probably attributable to the fact that the extracellular polymeric substances were attached to the surfaces of microorganisms, the population of which was similar in the two wwt cases, as it results from their MLSS values. As it is known, EPSs contain various types of organic macromolecules and mainly proteins and carbohydrates. They are found on the surfaces or in the interior of microbial aggregates and they are highly related to the biological kinetics affected by the influent wastewater and sludge properties. [7] They present a three-dimensional and highly hydrated structure as a result of the complicated interactions within their subcomponents. EPS contain charged and non-polar groups presenting amphoteric behavior [8].

### 3.3. Study of the Membrane Fouling Mechanism for Synthetic and Domestic Wastewater

**Figure 7:**
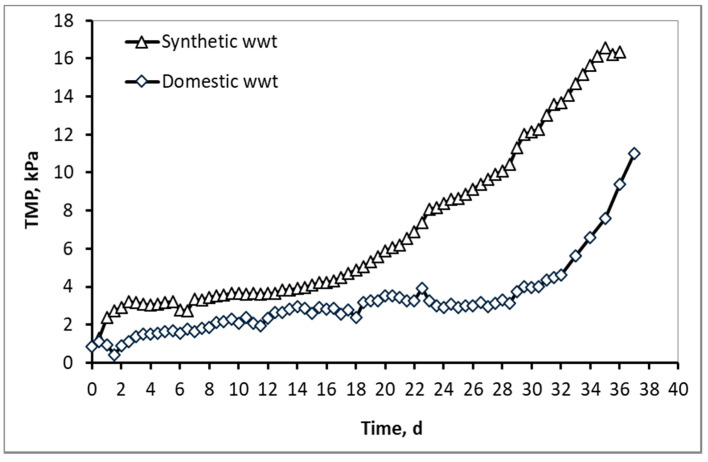
Typical TMP graphs as a function of MBR operation time for synthetic versus domestic wastewater. Flux for synthetic wwt = 16.4 Lmh, flux for domestic wwt = 20.9 Lmh. For both MBRs temperature = 24 ± 2 °C and DO in both ATs =2.5 ± 0.5 mg/L.

Regarding the membrane fouling, which is associated with the critical membrane filtration flux (Paragraph 2.2) and the TMP graphs (Figure 7), it is clear that the membrane fouling is more severe in the synthetic wwt compared to the domestic wwt. More specifically, the critical flux was 16.4 Lmh for synthetic wastewater and 20.9 Lmh for domestic wastewater, and therefore the difficulty of filtering synthetic wwt compared to domestic is evident. At the same time, even though synthetic wwt was filtered at a lower flow rate, it was observed that the transmembrane pressure (TMP) was constantly higher than the domestic wwt throughout the operation of the MBRs. Additionally, the TMP at 36 operating days reached 16 kPa, compared to 11 kPa for the domestic wwt.

**Figure 8:**
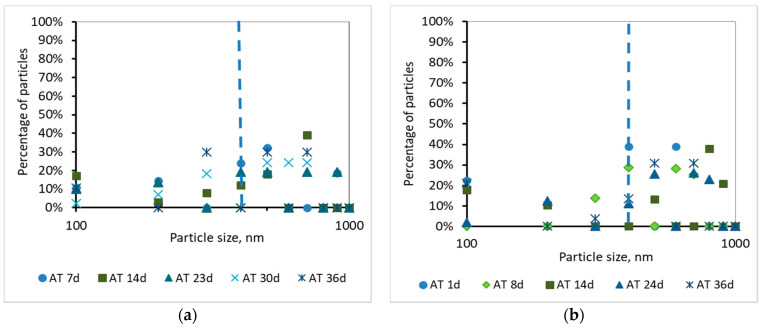
Percentage of particles smaller than 1000 nm for a sample from the aeration tank (AΤ) as a function of MBR operation time (**a**) for synthetic versus (**b**) domestic wastewater.

**Figure 9:**
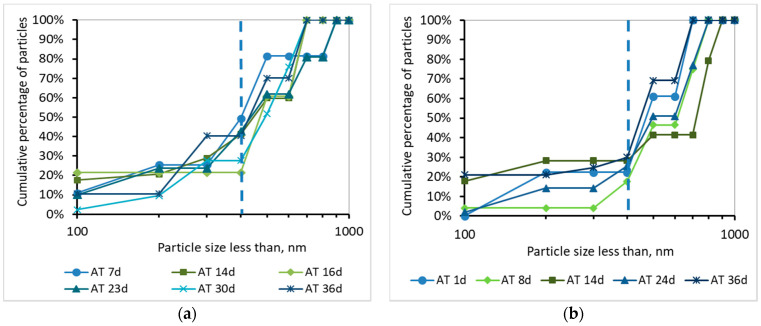
Cumulative percentage of particles smaller than 1000 nm for a sample from the aeration tank (AΤ) as a function of MBR operation time (**a**) for synthetic versus (**b**) domestic wastewater.

**Figure 10:**
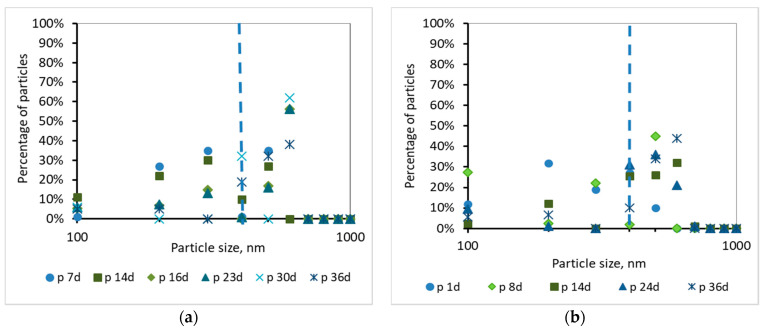
Percentage of particles smaller than 1000 nm for samples of the permeate (p) as a function of MBR operation time (**a**) for synthetic versus (**b**) domestic wastewater.

**Figure 11:**
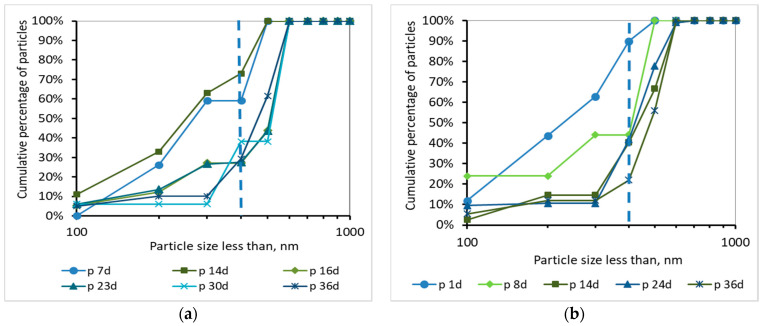
Cumulative percentage of particles smaller than 1000 nm for samples of the permeate (p) as a function of MBR operation time (**a**) for synthetic versus (**b**) domestic wastewater.

Τhe development of the TMP can be justified by the percentage and the cumulative percentage of particles smaller than 1000 nm, before and after membrane filtration, presented at Figure 8, Figure 9, Figure 10 and Figure 11. According to Figure 8, the particles smaller than 400 nm (membrane pore size) in the case of synthetic wwt prevail over the domestic wwt, whereas the particles greater than 400 nm are more in the case of domestic wwt. This conclusion is further supported by the cumulative percentage of particles presented in Figure 9, where, in the case of the synthetic wwt, 21–49% of particles were smaller than 400 nm (membrane pore size), whereas for the domestic wwt 15–30% of particles were smaller than 400 nm. Therefore, the colloids smaller than 400 nm in the activated sludge of the synthetic wastewater were about 20% more than in domestic wastewater. The greater production of small colloids in synthetic wwt may be attributed to the lower F/M loading (0.16 ± 0.03 g COD/g MLSS/d), compared to domestic wwt (0.24 ± 0.03 g COD/g MLSS/d). The low F/M ratio implies that a great number of microorganisms have little food available to consume, thereby potentially resulting in dead constituents that may have the form of small colloids.

According to Figure 10 and Figure 11, and the abovementioned Figure 7, the following conclusions were reached. For both wastewater types, it is concluded that the colloids passing through the membrane pores were gradually deposited and aggregated within the membrane pores, and then exited as aggregates in the permeate, causing irreversible membrane fouling [17]. Furthermore, it is concluded, judging from the results, that the composition of synthetic wastewater results in faster deposition and aggregation of colloids in the membrane pores, resulting in more severe membrane fouling compared to domestic wwt. These results may be attributed to colloids with size < 400 nm, which were more than 20% in the case of synthetic wwt, as mentioned above.

These conclusions are verified by the following considerations. In synthetic wwt, at 14 d of MBR operation, 73% of colloids exited at the filtrate were smaller than 400 nm, and the TMP was equal to 4 kPa. Subsequently, at 23 d of operation, only 27% of the colloids were smaller than 400 nm, whereas TMP was 8 kPa. After 23 d of operation, the remaining 73% of the colloids had been exited as much as larger aggregates. Therefore, the TMP increased as the aggregates were trying to exit from the fouled membrane pores in the permeate.

On the other hand, in domestic wwt, the membrane fouling was lower at 24 d, with TMP = 3 kPa. During this day, the passing colloids with size less than 400 nm were significantly more than those of the synthetic wwt, making up 40% of the total amount. Thus, in the case of domestic wwt, the deposition and aggregation of colloids within the membrane pores was evolved at a much lower rate.

According to the research of Banti et al. (2018) [17]—of the same research team—an identical hydrophilic membrane was observed under a scanning electron microscope (SEM), whereby the membrane surface was shown to contain pores of size ranging from 300 to 1200 nm. In particular, 50% of the pores presented diameters of 310 nm, whereas 35% of them were found to have diameters of 540 nm. Taking into account that SEM can give results only regarding the apparent surface pore size, the membranes are filter media in depth, and their real pore sizes are actually smaller than the corresponding pore sizes of their surfaces [27], it was concluded that the real diameters of pores of a fouled membrane are preferably evaluated by the sizes of the constituents excreted in the filtrate instead of the SEM images. Following these findings, in this study the mechanism of fouling of membrane pores is described in detail in the Figure 10 and Figure 11 according to the sizes of the components that enter and excrete from the membrane pores in keeping with the DLS measurements.

## 4. Discussion

The effluents of both MBRs are of high quality, as expected for the MBR units [5]. Regarding the SMP growth in the two wwt types, it resulted that the domestic wwt had 5.5 times more SMP proteins and 11 times more SMP carbohydrates compared to the synthetic wwt. The increased values of SMP proteins in the domestic wwt are attributed to the increased F/M values, and to the lower COD/N ratio in domestic wwt, compared to the synthetic wwt. At this point it should be noted that the differences in F/M and COD/N ratio values between the two wwt types were deliberate, in order to differentiate the SMP values and draw conclusions regarding their importance in membrane fouling. The increase of the substrate is followed by the improvement of bacterial metabolism [28] resulting in increased metabolism products, such as SMP. Furthermore, the SMP abundance in real domestic wwt may be attributed to its slower biodegradability, while synthetic wastewater is more readily biodegradable. Therefore, microorganisms consume the “substrate” of the synthetic wwt more readily compared to the domestic, and then once their food is exhausted, they also consume the SMP [23]. The experimental results of the abundance of the SMP proteins and carbohydrates in the domestic wwt, compared to the synthetic, are in agreement with those of other research that studied either domestic wastewater from a corresponding wastewater treatment plant or synthetic wastewater of the same composition [1,17,29]. However, they come in contrast to other studies [18], due to the diversity that occurs in municipal wastewater of different towns/countries (the domestic wastewater came from a wastewater treatment plant in France in that study), the different composition of synthetic wastewater (mass ratios of 2.1 C_6_H_12_O_6_, 1.0 (NH_4_)_2_SO_4_, 0.2 KH_2_PO_4_, 0.4 NaHCO_3_, 0.1 MgSO_4_ and 0.02 CaCl_2_), the different operational conditions (MLSS = 8.5 mg/L) and the different SMP extraction method (4000 g centrifugation, 20 min, 4 °C). It should be noted that despite the fact that a tubular ceramic membrane (ultrafiltration) made with ZrO_2_–TiO_2_ was used during that research, it was not the type and nature of the membrane that affected differently the SMP production and characteristics, but on the contrary it was the feedwater characteristics, the operational conditions and the SMP extraction method [8], as mentioned above.

In contrast to the SMP, the LBEPS and TBEPS—either in the form of proteins or in the form of carbohydrates—ranged at about the same levels for both synthetic and domestic wwt. This result is probably attributed to the fact that the extracellular polymeric substances are attached to the surfaces of microorganisms, the population of which was similar in the two wwt cases and equal to 4.500 ± 500 mg/L. Differences are, also, observed in the SMP and LBEPS proteins/carbohydrates ratios for the two types of wastewater, with synthetic wastewater showing higher values. This is attributed to the fact that the MBR unit that processed the synthetic wwt had a longer hydraulic retention time compared to the one that processed the domestic wwt. High hydraulic retention time is connected to a reduction of carbohydrates in the mixed liquor of synthetic wwt, as the microorganisms have time to digest their food, and specifically the carbohydrates that are more easily biodegradable compared to the proteins. Furthermore, according to the particle size distribution in the mixed liquor, it is concluded that the colloids with a size of less than 400 nm in the activated sludge of synthetic wwt were more than 20% compared to the corresponding value of the domestic wwt.

To summarize, the synthetic wwt had low SMP concentration and high colloidal composition, whereas the domestic wwt contained high SMP concentration and low colloidal synthesis. In terms of membrane fouling, as measured by the critical membrane filtration flux but also according to the TMP graphs, it is clear that the membrane fouling was more severe in the synthetic wwt compared to the domestic wwt. Specifically, the TMP at 36 days reached 16 kPa for the synthetic wwt and 11 kPa for the domestic. Therefore, synthetic wwt—despite its low concentration of SMP—caused severe membrane fouling compared to domestic, a result that is attributed to the increased concentration of colloids. The colloids smaller than membrane pore size can either adhere to a membrane surface or penetrate membrane pores or clog them [17], significantly deteriorating the irreversible membrane fouling (or not). Consequently, on the one hand, the quantity of colloids—and possibly their special characteristics—participate in conjunction with the SMP in membrane fouling, but on the other hand, judging from the results, they play a more decisive and important role in membrane fouling compared to the SMP.

According to Christensen et al. (2018) [30] who separated the activated sludge into floc/colloid, colloid/solute and solute fractions and observed the flux behavior, it was concluded that small colloid particles increase the hydraulic resistance, and therefore reducing fouling requires lowered concentrations of colloids and solutes. Moreover, according to other researchers [31] the rapid increase in TMP was attributed to organic solutes and colloids in the feed wastewater. Finally, as a result of this work, the results of other researchers are verified, and a new, innovative conclusion is also proven, which is the significantly higher contribution of colloids in membrane fouling compared to the SMP.

Moreover, for both wastewater types, it is concluded that the colloids passing through the membrane pores were gradually deposited and aggregated within the membrane pores and then exited as aggregates in the permeate. Therefore, the conclusion of Banti et al. (2018) [17] that has so far been confirmed only for domestic wastewater, has now been extended to other types of wastewater too. According to this conclusion, irreversible membrane fouling is attributed to the gradual deposition and aggregation of colloids within the membrane pores. It is furthermore concluded that the composition of synthetic wastewater results in faster deposition and aggregation of colloids in the membrane pores, resulting in more severe membrane fouling compared to domestic wwt. These results may also be attributed to the fact that the very small colloids with a size of <400 nm were more abundant (at about 20%) in the case of synthetic wwt, as mentioned above.

## 5. Conclusions

During this study, two lab-pilot MBRs were used to treat two different wastewater types, with the aim of studying the combined effect of colloids and soluble microbial products (SMP) on membrane fouling. In the first case, the synthetic wwt had deliberately low SMP concentration and high colloidal composition, whereas in the second case, the domestic wwt contained high SMP concentration and low colloidal synthesis. A COD removal of 97% and 95% for synthetic and domestic wastewater, respectively, was observed, whereas the nitrification process showed an efficiency of 100%. The lower efficiency of the domestic MBR in COD removal is mainly attributed to the non-biodegradable organic compounds of domestic wastewaters. According to the results, the domestic wwt had 5.5 times more SMP proteins and 11 times more SMP carbohydrates compared to the synthetic wwt. The higher concentration of SMP in domestic wwt is attributed to the higher F/M ratio (0.24 ± 0.03 g COD/g MLSS/d) and lower residence time (10.9 h) in domestic MBR, in comparison to synthetic wwt’s corresponding values of 0.16 ± 0.03 g COD/g MLSS/d and 13.9 h. Τhe increased values of SMP proteins in domestic wwt, equal to 82 ± 20 mg/L, are related to the lower COD/N ratio, while the relatively lower concentrations of carbohydrates, equal to 20 ± 9 mg/L, (compared to SMP proteins) are attributed to better assimilation by the microorganisms. Moreover, the synthetic wastewater had 20% more colloids (<400 nm, membrane pore size) in the mixed liquor than the domestic. The TMP at 36 operating days reached 16 kPa for synthetic wwt and 11 kPa for domestic wwt, from which we can conclude that the synthetic wwt caused increased and more severe membrane fouling. Moreover, for both wastewater types it is concluded that the colloids passing through the membrane pores were gradually deposited and aggregated within the membrane pores, and then exited as aggregates in the permeate, causing irreversible membrane fouling. Finally, synthetic wwt, despite its low concentration of SMP, caused severe membrane fouling compared to domestic, a result that is attributed to the increased concentration of colloids. Consequently, on the one hand, the quantity of colloids and possibly their special characteristics participate in conjunction with the SMP in membrane fouling, but on the other hand they play a more decisive role in membrane fouling compared to the SMP.

## Figures and Tables

**Table 1:** Specifications of the hydrophilic membrane.

Membrane configuration	A4 flat sheet (Type H 203, Kubota Corporation)
Base of membrane sheet	PET
Filtration material of membrane sheet	Chlorinated polyethylene (CPE)
Nominal pore size	0.4 μm
Effective membrane area	0.11 m^2^
Clean water initial permeate flow	7.2 L/h

**Table 2:** Physicochemical characteristics of influent and effluent for synthetic and domestic wastewater.

Parameters	Synthetic wwt		Domestic wwt	
	Influent	Effluent	Influent	Effluent
COD, mg/L	447 ± 68	14 ± 4.0	496 ± 71	26 ± 6.4
NH_4_-N, mg/L	28 ± 11	0.02 ± 0.01	34 ± 5.3	0.02 ± 0.02
NO_3_-N, mg/L	0.16 ± 0.12	26 ± 10	0.47 ± 0.13	34 ± 21
Total N, mg/L	46 ± 12	30 ± 8	75 ± 11	44 ± 19
COD/Total N	9.7	-	6.6	-
F/M, g COD/g MLSS/d	0.16 ± 0.03		0.24 ± 0.03	
Retention time ^1^, h	13.9		10.9	

^1^ Incorporates the volume of aeration (20 L) and membrane (5 L) tank.

**Table 3:** Soluble microbial products (SMP) and extracellular polymeric substances (EPS) comparison in synthetic and domestic wastewater.

SMP & EPS Concentrations	Synthetic Wastewater			Domestic Wastewater		
	Average	Min	Max	Average	Min	Max
SMP proteins, mg/L	15 ± 9	5	27	82 ± 20	51	105
LBEPS proteins, mg/g VSS	12 ± 6	4	20	13 ± 9	1	25
TBEPS proteins, mg/g VSS	39 ± 6	29	46	38 ± 9	25	55
SMP carbohydrates, mg/L	1.8 ± 1.1	0.5	5	20 ± 9	5	31
LBEPS carbohydrates, mg/g VSS	1.3 ± 0.5	0.5	2	2 ± 1.6	0.0	5
TBEPS carbohydrates, mg/g VSS	6 ± 2	3	10	8 ± 3	4	14

**Table 4:** Comparison of proteins/carbohydrates ratio for SMP and EPS between synthetic and domestic wastewater.

Proteins/Carbohydrates Ratio	Synthetic Wastewater	Domestic Wastewater
SMP Proteins/Carbohydrates	9 ± 4	4 ± 2
LBEPS Proteins/Carbohydrates	10 ± 3	5 ± 2
TBEPS Proteins/Carbohydrates	6 ± 3	6 ± 2

## References

[1] Banti D.C., Karayannakidis P.D., Samaras P., Mitrakas M.G. (2017). An innovative bioreactor set-up that reduces membrane fouling by adjusting the filamentous bacterial population. J. Memb. Sci..

[2] Mo J., Yang Q., Zhang N., Zhang W., Zheng Y., Zhang Z. (2018). A review on agro-industrial waste (AIW) derived adsorbents for water and wastewater treatment. J. Environ. Manag..

[3] Zhang W., Jiang F. (2019). Membrane fouling in aerobic granular sludge (AGS)-membrane bioreactor (MBR): Effect of AGS size. Water Res..

[4] Chen W., Mo J., Du X., Zhang Z., Zhang W. (2019). Biomimetic dynamic membrane for aquatic dye removal. Water Res..

[5] Lin H., Gao W., Meng F., Liao B.-Q., Leung K.-T., Zhao L., Hong H. (2012). Membrane Bioreactors for Industrial Wastewater Treatment: A Critical Review. Crit. Rev. Environ. Sci. Technol..

[6] Gkotsis P., Banti D., Peleka E., Zouboulis A., Samaras P. (2014). Fouling Issues in Membrane Bioreactors (MBRs) for Wastewater Treatment: Major Mechanisms, Prevention and Control Strategies. Processes.

[7] Wang Z., Han X., Ma J., Wang P., Mei X., Wu Z. (2013). Recent advances in membrane fouling caused by extracellular polymeric substances: A mini-review. Desalin. Water Treat..

[8] Lin H., Zhang M., Wang F., Meng F., Liao B.Q., Hong H., Gao W. (2014). A critical review of extracellular polymeric substances (EPSs) in membrane bioreactors: Characteristics, roles in membrane fouling and control strategies. J. Memb. Sci..

[9] Mesquita P.L., Aquino S.F., Xavier A.L.P., Da Silva J.C.C., Afonso R.C.F., Queiroz Silva S. (2010). Soluble microbial product (smp) characterization in bench-scale aerobic and anaerobic cstrs under different operational conditions. Braz. J. Chem. Eng..

[10] Meng F., Zhang S., Oh Y., Zhou Z., Shin H.S., Chae S.R. (2017). Fouling in membrane bioreactors: An updated review. Water Res..

[11] Tang S., Wang Z., Wu Z., Zhou Q. (2010). Role of dissolved organic matters (DOM) in membrane fouling of membrane bioreactors for municipal wastewater treatment. J. Hazard. Mater..

[12] Guo W., Ngo H.H., Li J. (2012). A mini-review on membrane fouling. Bioresour. Technol..

[13] Frolund B., Palmgren R., Keiding K., Nielsen P.H. (1996). Extraction of extracellular polymers from activated sludge using a cation exchange resin. Water Res..

[14] Wang X.M., Li X.Y., Huang X. (2007). Membrane fouling in a submerged membrane bioreactor (SMBR): Characterisation of the sludge cake and its high filtration resistance. Sep. Purif. Technol..

[15] Yiantsios S.G., Karabelas A.J. (1998). The effect of colloid stability on membrane fouling. Desalination.

[16] Lim A.L., Bai R. (2003). Membrane fouling and cleaning in microfiltration of activated sludge wastewater. J. Membr. Sci..

[17] Banti D.C., Samaras P., Tsioptsias C., Zouboulis A., Mitrakas M. (2018). Mechanism of SMP aggregation within the pores of hydrophilic and hydrophobic MBR membranes and aggregates detachment. Sep. Purif. Technol..

[18] Villain M., Bourven I., Guibaud G., Marrot B. (2014). Impact of synthetic or real urban wastewater on membrane bioreactor (MBR) performances and membrane fouling under stable conditions. Bioresour. Technol..

[19] Zhou L., Zhang Z., Meng X., Fan J., Xia S. (2014). New insight into the effects of Ca(II) on cake layer structure in submerged membrane bioreactors. Biofouling.

[20] Le-Clech P., Chen V., Fane T.A.G. (2006). Fouling in membrane bioreactors used in wastewater treatment. J. Memb. Sci..

[21] Van der Marel P., Zwijnenburg A., Kemperman A., Wessling M., Temmink H., van der Meer W. (2009). An improved flux-step method to determine the critical flux and the critical flux for irreversibility in a membrane bioreactor. J. Memb. Sci..

[22] Hwang B.K., Kim J.H., Ahn C.H., Lee C.H., Ra Y.H. (2010). Effect of disintegrated sludge recycling on membrane permeability in a membrane bioreactor combined with a turbulent jet flow ozone contactor. Water Res..

[23] Wang Z., Mei X., Ma J., Grasmick A., Wu Z. (2013). Potential Foulants and Fouling Indicators in MBRs: A Critical Review. Sep. Sci. Technol..

[24] DuBois M., Gilles K.A., Hamilton J.K., Rebers P.A., Smith F. (1956). Colorimetric Method for Determination of Sugars and Related Substances. Anal. Chem..

[25] Hartree E.F. (1972). Determination of protein: A modification of the lowry method that gives a linear photometric response. Anal. Biochem..

[26] Clesceri L., Greenberg A.E., Eaton A.D. (1967). Standard methods for the examination of water and wastewater. Health Lab. Sci..

[27] Wang L.K., Chen J.P., Hung Y.T., Shammas N.K. (2011). Membrane and desalination technologies. Handbook of Environmental Engineering.

[28] Lobos J., Wisniewski C., Heran M., Grasmick A. (2008). Sequencing versus continuous membrane bioreactors: Effect of substrate to biomass ratio (F/M) on process performance. J. Memb. Sci..

[29] Kampouris I.D., Karayannakidis P.D., Banti D.C., Sakoula D., Konstantinidis D., Yiangou M., Samaras P.E. (2018). Evaluation of a novel quorum quenching strain for MBR biofouling mitigation. Water Res..

[30] Christensen M.L., Niessen W., Sørensen N.B., Hansen S.H., Jørgensen M.K., Nielsen P.H. (2018). Sludge fractionation as a method to study and predict fouling in MBR systems. Sep. Purif. Technol..

[31] Jiang T., Kennedy M.D., van der Meer W.G.J., Vanrolleghem P.A., Schippers J.C. (2003). The role of blocking and cake filtration in MBR fouling. Desalination.

